# Nourishing the brain on deep space missions: nutritional psychiatry in promoting resilience

**DOI:** 10.3389/fncir.2023.1170395

**Published:** 2023-08-17

**Authors:** Nihar N. Pathare, Flavia Fayet-Moore, Jennifer A. Fogarty, Felice N. Jacka, Philip Strandwitz, Gary E. Strangman, Dorit B. Donoviel

**Affiliations:** ^1^Center for Space Medicine, Baylor College of Medicine, Houston, TX, United States; ^2^Nutrition Research Australia, Sydney, NSW, Australia; ^3^Department of Medicine, Baylor College of Medicine, Houston, TX, United States; ^4^Translational Research Institute for Space Health (TRISH), Houston, TX, United States; ^5^Food and Mood Centre, Institute for Mental and Physical Health and Clinical Translation (IMPACT) Strategic Research Centre, Deakin University, Geelong, VIC, Australia; ^6^Department of Psychiatry, The University of Melbourne, Parkville, VIC, Australia; ^7^Holobiome, Inc., Boston, MA, United States; ^8^Neural Systems Group, Division of Health Sciences and Technology, Massachusetts General Hospital, Harvard Medical School and Harvard-MIT, Charlestown, MA, United States; ^9^Department of Psychology, Harvard University, Cambridge, MA, United States; ^10^Department of Pharmacology and Chemical Biology, Baylor College of Medicine, Houston, TX, United States

**Keywords:** space, astronaut, mental-health, psychiatry, nutrition, food, microbiome, probiotics

## Abstract

The grueling psychological demands of a journey into deep space coupled with ever-increasing distances away from home pose a unique problem: how can we best take advantage of the benefits of fresh foods in a place that has none? Here, we consider the biggest challenges associated with our current spaceflight food system, highlight the importance of supporting optimal brain health on missions into deep space, and discuss evidence about food components that impact brain health. We propose a future food system that leverages the gut microbiota that can be individually tailored to best support the brain and mental health of crews on deep space long-duration missions. Working toward this goal, we will also be making investments in sustainable means to nourish the crew that remains here on spaceship Earth.

## Nourishing the brain in deep space

Deep space missions will test crews in unprecedented ways. The hazards of this hostile environment include unshielded ionizing radiation, prolonged isolation from loved ones, extreme physical confinement, a distance from Earth resulting in major communication delays and inability to resupply food and other essentials, physiological challenges of microgravity, and the psychological toll of living in a highly complex spacecraft susceptible to catastrophic engineering or human failures, with the ever-present knowledge that no one from the “outside” can help.

These hazards of deep space human exploration require mitigation if we are to protect the health and performance of the crew, demanding powerful, holistic, and feasible strategies to foster mental and physical resilience. Safeguarding brain health is of paramount importance to ensure cognitive performance and emotional regulation throughout the mission. Scientific consensus linking food and brain health propels our position that the deep space food system should offer a powerful tool for promoting resilience. This includes addressing the gut microbiome composition, which modulates mood, hormonal balance, and brain function and adaptation through neuronal and inflammatory effects (Berding et al., 2021; Shi et al., 2022). One such target is a form of bidirectional communication known as the gut-brain axis that depends on direct nerve firing, the release of hormones, and other bioactive molecules. It also offers a challenge: create bespoke diets contributing to diverse microbiomes, individually tailored to optimize physical and mental health.

In this Perspective, we describe the unique combination of spaceflight-associated challenges to brain health, the evolution of our current spaceflight food system, and the challenges associated with developing a deep-space food system. We then discuss the evidence base for nutritional psychiatry and break down the nutritional and psychoactive components of food. Finally, we review microbiome-based approaches to promote brain health and offer a vision for a future food system that aims at much more than just providing nutritional minimums but instead promotes health and resilience in humans wherever they explore.

## The brain in space

Spaceflight’s effects on human brain health range from cellular to social, including through neuroinflammation, sleep disruption, impacted cognitive performance, and psychosocial issues including poor team dynamics.

Deep space missions inherently involve dramatic increases in exposure to highly charged and energetic (HZE) ionizing radiation, which affects cellular function. Dose-relevant radiation studies have revealed direct effects on neuronal tissue (Raber et al., 2016; Jandial et al., 2018), functional decrements (Vlkolinsky et al., 2010; Blackwell et al., 2022), as well as oxidative stress and neuroinflammation (Zwart et al., 2021; Holley et al., 2022; Miller et al., 2022; Verma et al., 2022). Neuroinflammation is in turn associated with a wide range of maladies—including neurodegenerative diseases such as Alzheimer’s, Parkinson’s, and Huntington’s diseases (Teleanu et al., 2022).

Astronauts experience a headward fluid shift in zero gravity which has been correlated with a decrease in the sensitivity of aroma perception which could contribute to a reduced daily caloric intake of 80 percent (Taylor et al., 2020). Spaceflight also poses challenges to the human sleep and circadian system, although the exact causes of such sleep disturbances remain unclear. In low-Earth orbit, astronauts sleep an average of 6 h/per night (Barger et al., 2014), which can significantly impair behavioral performance (Jewett et al., 1999). This occurs even though sleep medications are used at a rate approximately ten times higher in space than on Earth (Putcha et al., 1999; Barger et al., 2014; Wotring, 2015). Significant sleep and circadian disruptions have also been observed in space confinement simulations such as Mars520 (Basner et al., 2013), low-/artificial-light settings (Phillips et al., 2019), and ground simulations of a 24.6-h Martian day (Nguyen and Wright, 2010), despite the use of countermeasures (Barger et al., 2012). Inadequate sleep duration or quality has been linked to suboptimal cognitive, behavioral, and physical performance (Van Dongen et al., 2003).

To date, studies have not demonstrated major disruptions in cognitive performance during spaceflight (Strangman et al., 2014), but important caveats remain. Nearly every cognitive performance study in spaceflight has had serious shortcomings; too few subjects, low sensitivity of measures, limited coverage of cognitive processes, as well as task variability that has prevented meta-analysis across studies are all common (Strangman et al., 2014). In one rare mission, a case-controlled human study involving detailed cognitive testing before, during, and after a 340-day spaceflight revealed diminished cognitive performance that extended for at least 6 months post-flight (Garrett-Bakelman et al., 2019). This unique long-duration flight still only represented 35–40% of the anticipated duration of a mission for landing on Mars (at ∼900 days). Intact cognitive performance in deep space missions thus remains a critical risk needing surveillance and mitigation.

Psychological health issues have occurred in spaceflight and analogs (Kanas and Manzey, 2008). These have included depression and anxiety (Stuster, 2010), as well as at least one suggestion of an in-flight and potentially behavioral-related emergency leading to an abrupt abort of the Soyuz T14-Salyut 7 mission (Morris, n.d.). Such behavioral health findings parallel reports on isolation studies on Earth (Lugg, 2005; Basner et al., 2013), and point to non-radiation-induced changes in brain health that result in behavioral health issues in settings that are far less resource constrained than spaceflight.

Finally, in addition to the impact on individuals, team performance challenges have arisen during spaceflight missions. In astronaut journals, the number of positive comments about team interaction decreases over the mission (Stuster, 2010). Reductions in team cohesion (e.g., subgrouping) have been reported in various analogs (Kanas et al., 2009; Basner et al., 2013; Tafforin et al., 2015), and communication with ground personnel decreases over time (psychological “closing”) (Gushin et al., 2012). A meta-analysis of various mission types indicated that each team had at least one conflict by 90 days into their mission (Bell et al., 2019). The increasing distance from Earth causes communication delays of up to 22 min each way, and a Mars mission will isolate the crew even more, exacerbating many of these challenges.

Various approaches used on the International Space Station (ISS) to avoid or mitigate psychological, behavioral, and team issues are dependent upon real-time communications (calling family/friends, interaction with flight psychologists and psychiatrists, special events with ground personnel), resupply (special foods, surprise deliveries), and free time to gaze and photograph the beauty of Earth (Earth-viewing: a prominent de-stressing activity). Deep space missions will not offer most of these approaches.

## The space food system

The US spaceflight food systems initially focused on the basic safety of consuming semi-solid foods. During the Mercury Program, once John Glenn demonstrated that humans could properly swallow applesauce, the focus was on providing engineered foods with appropriate calories and basic nutrients. This approach continued through the Apollo program with the addition of some food variety, but the system was limited to pastes or dehydrated meats, fruits, and vegetables (Perchonok and Bourland, 2002). Based on previous crew feedback regarding the importance of palatability, Skylab further improved the space exploration food system (Kerwin and Seddon, 2002). Research and development of the food system continued during the shuttle and ISS eras, and as missions extended to several months, the food system became more complex. Crews continued to highlight the criticality of the taste and texture of food to the overall success of missions. This criticality is punctuated by the nearly total lack of *in situ* food sources, complete reliance on Earth, and the need for the food system to be implementable in a closed, small, and constrained habitat.

The food requirements for a deep space Mars class exploration mission, 18–36 months, will surpass any experience that is equivalent in resource limitations to date. While there have been expeditions to remote places on Earth, many parameters such as total duration, time in deep space transit, time on a distant planet, number of spacewalks, etc., remain relatively undefined so far and present unique challenges in designing an ideal food system. We need to understand what a diverse crew will need, to endure the extreme spaceflight duration, and meet performance expectations. Even though the space food system has an essential role in the crew’s survival, it is also part of an operational environment that has significant competition with other systems for mass, power, and volume. Also, it must not introduce additional risks. This means that the system must accomplish many critical functions with the lowest impact on the overall vehicle and mission architecture. This is incredibly challenging when the humans, vehicle design, and mission operations are only generally described based on experience and models. The food system is then guided only by known and emerging requirements and best practices.

Earth-based human food requirements are adjusted for spaceflight based on the mission parameters and the anticipated environmental impacts on the human. Mission parameters include the duration of the mission, the need to pre-deploy food, the ability to resupply food, to provide fresh foods during the mission timeframe (growing crops, cultured protein sources, etc.), and the crew performance requirements (physical and cognitive). The mission parameters determine the duration of preservation and the need to supplement micronutrients or for augmentation of the food system with freshly grown or cultured components. Each parameter must be assessed for safety and stability. All foods, whether processed and stored, or freshly grown/cultured, need to be safe to eat, nutritionally adequate, and palatable, meeting all the crew’s needs. Each component of the food system must be robust so that the crew can rely on it. There is a long history of preserved foods that address reliability, but their lifespan is inadequate for the ∼5-year duration required to support the Mars exploration class mission because of the requirement to pre-deploy food to the planet’s surface well in advance of the arrival of the crew. We also know that the preserved food component is inadequate to address micronutrient requirements throughout the period that the food system would be stored, and a Mars crew would consume it (Cooper et al., 2011, 2017; Perchonok et al., 2012; Douglas et al., 2020). Decrements in the food system used today include the degradation of labile vitamins essential for health as well as stability, palatability of the meal components, and adequate variety of food choices. Therefore, investment in the development of the capability to grow fruits and vegetables during spaceflight has increased significantly, as has the emerging capability to culture protein sources for spaceflight, while the technology and acceptability are maturing on Earth. New information on the likely critical components of foods beyond macro and micronutrients (i.e., bioactive compounds, fermentation products, bacteria themselves) is prompting consideration of other forms of augmentation that may yield improvements for nutrition, flavor and texture, and preservation.

While we have learned much about providing a safe, reliable, diverse, and palatable food system for low Earth orbit, there is still much work to be done to explore successful ways to drive a more sustainable food future as well as to enhance the current space food system that is based heavily on preserved foods with fresh and nutrient-rich foods frequently resupplied from Earth. The food system of the future must not only meet the caloric and nutritional needs of the astronauts but also provide psychological resilience and prevent and mitigate the effects of spaceflight stressors.

## Food components critical for overall nutrition and brain health

Nutrition is one of the top three lifestyle factors that influence disease initiation and propagation in humans on Earth (Afshin et al., 2019). Its role is particularly paramount in space, where spaceflight hazards challenge the body and mind in unprecedented ways (Zwart et al., 2021). Foods and nutrition components required to maximize brain and cognitive function for astronauts on ISS or private citizens visiting commercial low Earth orbit destinations are likely to significantly differ from those that will be required on deep space missions. An adult’s brain consumes significantly more energy from glucose (20%) than expected for its size (2%) for neural functioning, and unlike muscles (which utilize glycogen), the brain does not have stored energy reserves and is always metabolically “on.” Hence, a balance of essential brain nutrients that includes long-chain omega-3 fatty acids, antioxidant vitamins (C and E), the b-vitamins including B12, and vitamin D, and the minerals iron, magnesium, and zinc together influence cognitive function and performance, brain development, oxygen transport, brain cell health, neurotransmitter function, brain structure, intercellular connections, and protect against oxidative stress (Tardy et al., 2020).

## The impact of food on mental and brain health

Diet is critical to mental as well as physical health and offers new opportunities for the prevention and treatment of mental health problems, as well as for optimizing mental and brain health under particularly challenging conditions such as space missions (Marx et al., 2017).

Diet quality is now understood as a modifiable, independent risk factor for depression, which itself represents the most common and burdensome mental health condition globally (Dash et al., 2016), experienced in both spaceflight and analogs. The associations between diet quality and depression risk are seen across the lifespan (Jacka et al., 2011, 2013a,b; Borge et al., 2017; Lassale et al., 2019). Importantly, evidence from randomized controlled trials shows that dietary interventions can treat even severe depressive illnesses (Jacka et al., 2017; Bayes et al., 2022) as well as improve depressive symptoms in non-clinical populations (Firth et al., 2019). While dietary habits vary across cultures, the fundamentals of what comprises a “healthy diet” associated with less depression are broadly similar (Opie et al., 2017)—plant foods (vegetables, fruits, wholegrain cereals, legumes, nuts, and seeds), healthy fats from seafood and plants, and a reduction in or avoidance of foods high in refined carbohydrates, salt, saturated and trans fats, and other additives, particularly those classified as “ultra-processed foods,” which are associated with poorer physical and mental health (Lane et al., 2021).

The multitude of mechanisms that link diet quality to mental health includes immune function, gut microbiota, brain plasticity, neurotransmitter and stress response systems, and gene expression (Marx et al., 2021). The range of mechanistic pathways implicated reflects the understanding that many aspects of mental illness do not just occur in the brain, but rather can be influenced by many bodily systems and processes. However, in adapting and optimizing brain health under extreme conditions such as those in deep space, animal studies suggest poor diet can impact cognition via inflammation and its effects on neurogenesis and hippocampal function is critical (Beilharz et al., 2015). The extensive evidence in this area is now supported by observational data in humans, wherein diet quality (Jacka et al., 2015) and dietary factors are clearly linked to the size of the hippocampus and other brain regions (Akbaraly et al., 2018; Croll et al., 2018). Moreover, intervention studies in humans have shown a rapid impact on cognitive function in young, healthy adults on western-type diets, high in saturated fats and added sugars (Stevenson et al., 2020). Highly relevant is the emerging evidence that a diet is enhanced for long-chain. omega-3 fatty acids and flavonoids via increased quantity and variety of plant foods and seafood led to better cognitive performance, among other parameters, in subjects undergoing closed chamber missions in the NASA Human Exploration Research Analog (HERA) (Douglas et al., 2022).

One of the key areas of investigation and promise relates to the human microbiota, which is now understood to influence all bodily systems and is, in turn, influenced by the brain and behavior. The largest number of microbes reside in the gut, where they break down elements of food that cannot be broken down by human enzymes, particularly plant fiber, and polyphenols. Current evidence links these dietary components, as well as fruits, vegetables, nuts, mono and polyunsaturated fatty acids (MUFA/PUFA), plant-based proteins, and—more widely—plant-focused and Mediterranean-style dietary patterns, to beneficial gut microbiota profiles, while animal proteins, saturated fatty acids, artificial sweeteners, and emulsifiers, as well as western diets more broadly, are linked to gut microbiota profiles associated with poor health outcomes (Berding et al., 2021). We discuss the microbiome further below.

## Examples of novel components of a deep space food system

The current food system on ISS relies on mostly prepackaged processed foods and frequent resupply with fresh foods. Resupply will not be an option on deep space missions. A trip to Mars would require some of the food to be prepositioned on the planet’s surface for many months in advance of the crew’s arrival. There are concerns about the nutritional stability of the food system for that long of a period. If we are to provide a healthy food system to support a Mars mission crew, we must go beyond providing the minimum nutritional and caloric requirements. There are promising new food sources and potentially bespoke approaches to utilizing microbes that can help safeguard health.

Algae, which belong to their own kingdom, and Protista, (neither plant nor animal), may help to meet the needs for long-chain omega-3 fatty acids including DHA (Winwood, 2013), the predominant fatty acid in the brain (Weiser et al., 2016). Further, they can capture and utilize carbon in closed-loop ecosystems and food systems, being an extraordinary carbon sink. There are over 500 species of algae, providing not only DHA, but other key micronutrients not typically found in plants (vitamin B12), and a non-animal source of protein and long-chain omega-3 fatty acids. Further, unlike plants, algae harvests need fewer inputs (i.e., some species such as *Chlorella pyrenoidosa* can obtain all nutrients from air and water) and some can be harvested daily (Han et al., 2017). Algae can grow in a microgravity environment, making them a suitable option in space. Fungi, and specifically edible mushrooms, are the only unfortified and non-animal source of dietary vitamin D and are grown in controlled environments. UVB-exposed mushrooms provide more than 100% of daily vitamin D requirements (Blumfield et al., 2020). Given UVB exposure via the skin is not feasible in deep space, innovation with both fungi and algae foods may allow for the development of nutrient-dense foods that provide a multitude of not only nutrients, but also their corresponding bioactive compounds, possibly combating some of the challenges (Tang et al., 2021) of creating palatable and nutritious food for long duration missions.

It is unreasonable to assume that we can provide astronauts with all the freshly grown foods needed to optimize health and performance in deep space; we will need to rely in a large part on prepackaged foods. There is increasing recognition of the importance of plant bioactive compounds (non-nutrient components) as a primary driver of health benefits beyond just the nutritional and caloric value (Rodriguez-Casado, 2016). About 20 years ago, research showed that flavonoids in apples explained most of their antioxidant properties, whereas vitamin C explained only 0.4% of the antioxidant activity (Liu, 2003). The health benefits associated with eating a rainbow of color (bioactive pigments) were recently proven (Blumfield et al., 2022). Further, color is a natural visual cue for food consumption (Pennock et al., 2023), and added color via bioactive compounds in food may provide further benefits related to food intake and wellbeing in deep space. There are more than 10,000 individual bioactive compounds in plant foods to date (Zhang et al., 2015), and enhancing their preservation in prepackaged foods for consumption in space should be a priority, especially given many have prebiotic properties linked to improved gut health (Plamada and Vodnar, 2021).

## The role of microbes in a deep space food system

Our microbiome is an entire second genome, one larger and more variable than our own. At the latest estimate, the human genome contains just under 20,000 protein-coding genes (Piovesan et al., 2019), with greater than 99.5% matching identity across our entire species. The average person’s microbiome is estimated to contain roughly one hundred times this number of genes (Qin et al., 2010). This functional power is further revealed by metabolomics, where nearly half of the metabolites found in blood are influenced by microbiome composition (Visconti et al., 2019). Most of our microbes live in our gastrointestinal tract, wherein a given person there typically exists hundreds of unique species of bacteria and lesser numbers of archaea, fungi, and bacteriophage (Huttenhower et al., 2012). These microbes are highly heritable in nature, some of which are passed to us from our mother (Grieneisen et al., 2021). The others reflect the people, experiences, and challenges that define our lives (Valles-Colomer et al., 2023).

Beyond controlling what our microbes are exposed to, programmed in our genome are mechanisms to influence and preserve the composition of our gut microbiome. These include our rich complement of immune cells, secretion of mucin glycans that feed commensal bacteria (Tailford et al., 2015), the release of bile salts (Schubert et al., 2017), lipocalin-2 (Flo et al., 2004), and antimicrobial peptides like beta-defensins (Ostaff et al., 2013) which largely target pathogens.

Unlike our genome, the microbiome can be disrupted by external factors, including antibiotics, infection, and a sub-optimal diet. Like any ecosystem, disruption of its key members can wreak havoc on its biology. In the past decade the importance of this balance has been increasingly highlighted, with the microbiome–and its disruption–having been linked to how we respond to diet (Kolodziejczyk et al., 2019) and drugs (Weersma et al., 2020), fight infection (Libertucci and Young, 2019) and cancer (Sepich-Poore et al., 2021), and even behave (Dinan et al., 2015). For example, in observational studies, individuals with major depressive disorder (MDD) have distinct gut microbiota profiles compared to healthy controls (Naseribafrouei et al., 2014; Jiang et al., 2015; Kelly et al., 2016; Zheng et al., 2016; Strandwitz et al., 2018; McGuinness et al., 2022).

The field is unraveling mechanistically how our microbes could be driving these observations, but there appears to be direct crosstalk between our microbes and our immune (Danping et al., 2020), endocrine (Neuman et al., 2015), and nervous (Fung et al., 2017) systems. This is mediated largely by secreted metabolites [such as neurotransmitters (Strandwitz, 2018)], protein-ligand interactions, and/or diet (Kolodziejczyk et al., 2019) or even drug interactions (Weersma et al., 2020). Beyond combating disease and making people live better lives here on Earth, unlocking the power of the microbiome could help humanity become multi-planetary. Manipulating the microbiome has the potential to combat spaceflight hazards, such as protecting against radiation damage (Guo et al., 2020), increasing resilience to stress (Berding et al., 2022), optimizing nutrition (Zeevi et al., 2015), and responding appropriately to medications (Gopalakrishnan et al., 2018). As such, there is a high potential in using our second genome as a “backup crew,” but to do so we need proper tools.

### Probiotics

At its core, the microbiome is microbes, and one way to change it is through the consumption of probiotics. Probiotics are defined as “live microorganisms that are intended to have health benefits when consumed or applied to the body” (Hill et al., 2014). As such, ideal processes to find probiotics include microbiome research, leveraging a combination of human cohorts and preclinical models, and identifying specific bacterial strains or combinations thereof which have a potential impact on a desired aspect of host biology. These strains are then put through manufacturing processes to ensure viability and stability, tested in placebo-controlled trials to generate confidence they are safe and do what we think they can, and then distributed either as a consumer product or drug with appropriate health claims, backed by strong data packages.

Unfortunately, while there are exceptions, many bacteria sold today have little or no data to support being a “probiotic.” Instead, the probiotic industry is dominated by products containing bacteria that are either absent from or are a very minor constituent of the adult gut microbiome, such as bacteria derived from the soil (e.g., *Bacillus*), the food/dairy industries (e.g., *Lactobacillus*, *Lactococcus*), and infants (*Bifidobacterium*). While these bacteria are largely safe, they represent a tiny fraction of the diversity and functional potential of the adult microbiome. To provide metrics, there are a few dozen species of bacteria sold as consumer probiotics, and very few are used as drugs or medical foods. In contrast, in each human, there are estimated to be several *hundred* unique bacterial species, and nearly 5,000 species in the collective human gut microbiome, most of which have no cultured representatives (Nayfach et al., 2019) (while each person has a few hundred species, not everyone has the same species).

The status is simple—today we do not have probiotics containing 99% of the diversity of what exists within us. This means we do not have a way to introduce these back into people who could benefit from their functions, except for fecal microbiome transplants. However, through advances in cultivation methods, the creation of comprehensive strain collections, and throughput mapping of host-microbiome interactions, the “next generation” of probiotics is coming.

### Prebiotics

A secondary means to modulate the microbiome is with prebiotics—often defined as “a fermented ingredient that results in specific changes in the composition and/or activity of the gastrointestinal microbiota, thus conferring benefit (s) upon host health” (Davani-Davari et al., 2019). In other words, something you eat impacts your microbes in a way beneficial to the system. Like probiotics, the field of prebiotics is still largely uncharted. Our food contains many components that our microbes use for their own metabolism—such as glycans, oligosaccharides, flavonoids, and peptides. Some have been purified and are commonly added to food or sold as supplements, like wheat dextrin, inulin, psyllium, and methylcellulose.

However, recent work has highlighted there is not a one size fits all approach. Different fibers (cellulose, inulin, pectin, and mixed fiber) elicited differential microbiome and metabolic signatures in mice with normalized microbiomes (Murga-Garrido et al., 2021). Similarly, in mice colonized with the microbiome from nine different human donors, significant variation of three different fiber sources (pea, orange, and barley bran) was seen amongst the different microbiome backgrounds (Delannoy-Bruno et al., 2021). This is further supported by a series of human studies monitoring the glycemic responses of individuals challenged with normalized diets—responses can vary widely. Some people had spikes with chocolate, some with bananas, and some with bread, an effect that appears to be driven by the composition of their microbiome (Zeevi et al., 2015; Korem et al., 2017). This highlights that we need a better understanding of the interactions between our microbes, their functions, and our food.

Historically, major challenges to advancing the next generation of prebiotics revolve around not knowing the chemical composition of our food, difficulties in producing more complex ingredients, and the absence of datasets directly interrogating how food and food ingredients impact the microbiome. It’s also important to reiterate that not all people have the same microbes, and in some cases, people simply will not have the microbes that certain prebiotics or diets are designed to support. This is a solvable problem if we can also deliver the right microbes in combination with the food. Reduced costs for microbiome sequencing, efforts to broadly annotate food chemistry, and better methods to track diet data leveraging digital health tools—coupled with screening efforts of food ingredients and human gut microbes—will pave the way for the future of precision prebiotics and nutrition.

## The future

A long-duration deep space mission where a combination of the major environmental hazards will challenge the physical and mental resilience of the crew will require a holistic approach to countermeasures. The food system can play a major role in safeguarding health and performance beyond just providing the minimum: nutrients and calories. It can be optimized using a variety of new food sources. Lab-grown animal products such as cultured meats and dairy could provide fresh supplies of sufficiently dense protein sources (Handral et al., 2022; Waltz, 2022). Algae and engineered plants that are optimized for growth in space can provide nutrients and bioactive compounds necessary for brain and physical health (Holly, 2019; Stewart et al., 2021).

In addition to novel methods to provide the daily requirements of macronutrients such as protein, fats, and carbohydrates, a deep space food system could be enhanced with an armamentarium of probiotics, representative of the diversity and functions we have evolved with, coupled with prebiotics to selectively support them. Such microbiome-modifying tools could be used to augment an astronaut’s biology and come with several advantages. Probiotics are user-friendly, self-replicating, and could be swallowed before launch and be designed to remain within the traveler’s gastrointestinal tract. By using appropriate prebiotics or dietary inputs (Abramson et al., 2019; Jimenez et al., 2019), these microbes can then become internalized bio-factories, producing essential nutrients otherwise difficult to attain with space diets, such as B and K vitamins (Hill, 1997), or other factors that modulate host systems relevant for a given stress. Leveraging synthetic biology, probiotics can also be engineered to produce and deliver drugs and other payloads (Canale et al., 2021; Russell et al., 2022), serve as biosensors for health risks (Chang et al., 2021), and even be improved to withstand various space-relevant stressors, like ionizing radiation (Jimenez et al., 2019). This provides a powerful technological tool—built on the brilliance of our own biology—that requires no change in the routine of the astronaut, and one that can be personalized ahead of the journey to space (Holmes et al., 2022).

The hazards of deep space missions demand nothing short of radical approaches to safeguarding the health of the crews that dare. The damage to the brain from ionizing radiation and the stressors of microgravity, isolation, and physical confinement can be at least partially mitigated by a robust and holistic food system, developed with state-of-the-art methods providing fresh proteins and vegetable products, and supplemented with bioactive compounds and personalized prebiotics and probiotics. As we build a novel deep-space food system, we also invest in sustainably feeding future generations on *terra firma*.

## Data availability statement

The original contributions presented in the study are included in the article/supplementary material, further inquiries can be directed to the corresponding author.

## Author contributions

DD organized the topic and recruited the authors. NP organized the manuscript and assisted with writing the manuscript. All authors wrote the portions of the manuscript.
